# Fabrication of Electromagnetically-Driven Tilted Microcoil on Polyimide Capillary Surface for Potential Single-Fiber Endoscope Scanner Application

**DOI:** 10.3390/mi9020061

**Published:** 2018-02-01

**Authors:** Zhuoqing Yang, Jianhao Shi, Bin Sun, Jinyuan Yao, Guifu Ding, Renshi Sawada

**Affiliations:** 1 National Key Laboratory of Science and Technology on Micro/Nano Fabrication, School of Electronics Information and Electrical Engineering, Shanghai Jiao Tong University, Shanghai 200240, China; yzhuoqing@sjtu.edu.cn (Z.Y.); sjh_timehao@163.com (J.S.); jyyao@sjtu.edu.cn (J.Y.); gfding@sjtu.edu.cn (G.D.); 2 Institute of Biomedical and Health Engineering, Shenzhen Institute of Advanced Technology, Chinese Academy of Science, Shenzhen 518055, China; 3 Graduate School of Systems Life Science, Kyushu University, Fukuoka 819-0395, Japan; sawada@mech.kyushu-u.ac.jp

**Keywords:** tilted microcoil, electromagnetically-driven, surface micromachining, polyimide capillary, MEMS

## Abstract

The design and fabrication of a Micro-electromechanical Systems (MEMS)-based tilted microcoil on a polyimide capillary are reported in this paper, proposed for an electromagnetically-driven single-fiber endoscope scanner application. The parameters of the tilted microcoil were optimized by simulation. It is proved that the largest driving force could be achieved when the tilt-angle, the pitch and the coil turns of the designed microcoil were 60°, 80 µm and 20, respectively. The modal simulation of the designed fiber scanner was carried out. The prototypes of the tilted microcoils were fabricated on the surface of polyimide capillary with 1 mm-diameter using our developed cylindrical projection lithography system. The dimensions of the two tilted microcoils were as follows: one was tilt-angle 45°, line width 10 ± 0.2 µm, coil pitch 78.5 ± 0.5 µm, and the other was tilt-angle 60°, line width 10 ± 0.2 µm, coil pitch 81.5 ± 0.5 µm. Finally, a direct mask-less electroplating process was employed to fabricate the copper microcoil with 15 µm thickness on the gold (Au) seed-layer, and the corresponding line width was expanded to 40 µm.

## 1. Introduction

Miniature devices have been developed extensively for medical and biological applications. Recently, to inspect the imaging within blood vessel and lactiferous ducts inside animal and human body, ultra-thin medical endoscopes have been developed by shrinking the overall diameter of the devices [1,2]. Conventional endoscopes mostly employ Charge Coupled Device (CCD) and Complementary Metal Oxide Semiconductor (CMOS) imaging sensors for image capture [3,4]. However, the main limitation of video-endoscopes is their diameters. Video-endoscopes <3 mm in diameter suffer from a reduced imaging quality when using conventional imaging technology. Consequently, a kind of flexible endoscope was ushered in by fiber-optic bundles [5,6]. It is also difficult to obtain high-resolution imaging within a thin fiber scope, due to the fact that the number of optical fibers is restricted. In order to improve the imaging quality, a smaller diameter, rapid-scanning and high-resolution optical scanner with a single fiber has recently been developed. 

Weber et al. [7] developed a single-fiber scanner, which employed a micro-mirror and a micro-motor to realize linear and rotated scanning, and the outer diameter of the assembled system was 2.75 mm. Hu et al. [8] proposed a single fiber coated with nickel magnetic gel, which was actuated by an external electromagnet. Weber et al. [9] also presented an endoscopic probe with a forward-looking piezoelectric fiber scanner with an outer diameter of 2.5 mm. Assadsangabi et al. [10] reported an optical fiber scanner using a micro rotary motor, which consisted of ferrofluid, permanent magnet and activation coil. Its final packaged outer diameter was 3 mm. Obviously, the packaged dimensions in the aforementioned designs were too large for some intra-corporeal applications in which the fiber scanner needs to fit in small diameter (<2 mm) organ ducts. In addition, Tadao et al. [2] reported an electromagnetically-driven ultra-miniature single-fiber scanner that was actuated by tilted microcoils fabricated on cylindrical substrates using a laser point exposure system. A microcoil with a tilt-angle of 45° and a coil pitch of 200 µm was successfully obtained, but scanning amplitude was limited because of the small driving force generated by a large pitch microcoil.

In this work, we present an ultra-thin electromagnetically-driven scanner fabricated by a novel Micro-electromechanical Systems (MEMS)-based fabrication method called cylindrical projection lithography, which is a new fabrication technology for manufacturing functional microstructures or transducers on the surface of cylindrical substrates as micro components in some biomedical tools. The developed cylindrical projection lithography method can be used for directly patterning tilted microcoil on the surface of a polyimide capillary as an electromagnetically-driven actuator. The microcoil patterns of photoresist film can be fabricated with different tilt-angles. More importantly, a smaller line width and coil pitch can be also realized. Thus, the fabricated microcoil would efficiently enhance the electromagnetically driven force, despite its miniature size. The corresponding fabrication process also includes spray-coating technology and a direct mask-less electroplating process on a microcoil pattern with a seed layer. 

We begin in the next section by describing the configuration and principle of the designed single-fiber scanner, followed by optimizing the related structural parameters. Subsequently, the cylindrical projection lithography technique and fabrication process are presented. Finally, the fabrication and characterization of the tilted microcoils are presented.

## 2. Design and Simulation 

### 2.1. Single-Fiber Scanner

**Figure 1:**
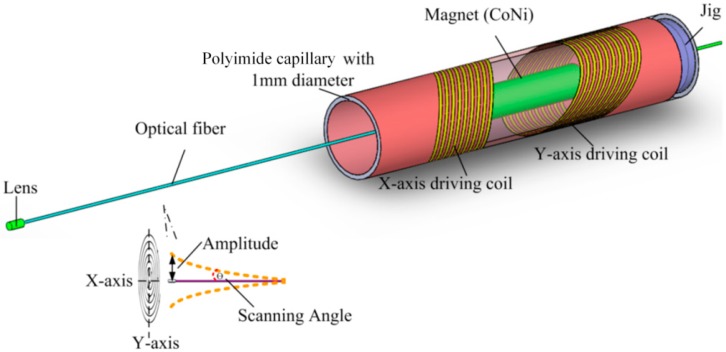
Sketch of proposed single-fiber scanner by electromagnetically driven tilted microcoil to which AC power is supplied.

To realize a large scanning amplitude in the narrow space of the human body, a novel electromagnetically driven MEMS-based single-fiber scanner has been designed, as illustrated in Figure 1. This fiber scanner is composed of a single-mode optical fiber, a collimator lens, a cylindrical magnet, a jig, and two tilted microcoils (X-axis driving coil and Y-axis driving coil). A 125 µm diameter optical fiber is positioned in the middle of the polyimide capillary using a jig with a small hole in it, which is manufactured by a 3D printer. A micro collimator lens is fused at the tip of the optical fiber, and a cylindrical magnet (Cobalt Nickel) is fixed on the fiber. Finally, two tilted microcoils are fabricated on the surface of the 1 mm diameter polyimide capillary with a wall thickness of 0.1 mm (SCHOTT MORITEX Corporation, Cambridge, UK). Its melting point and dielectric constant are 400 °C and 3.4 respectively. When AC power is supplied to the tilted microcoil, the fiber will vibrate continuously in period under the effect of magnetic force torque. By supplying AC power to both of the driving coils with a 90° phase shift, the fiber can scan in two dimensions. When the frequency of the applied AC is the same as that of the fiber with magnet, the whole structure will continuously scan with the largest amplitude scanning angle.

### 2.2. Design of Tilted Microcoil

**Figure 2:**
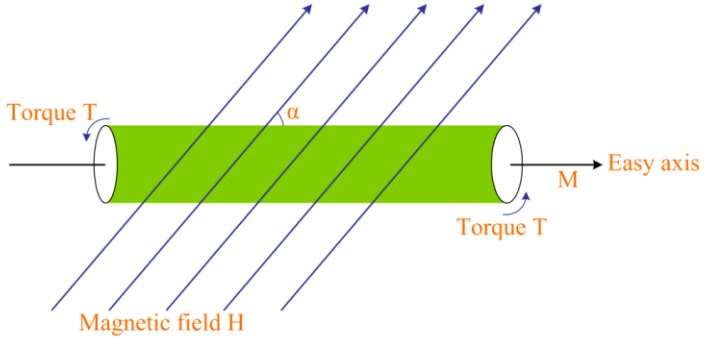
Schematic of torque calculation under tilt magnetic field.

Theoretically, when a soft magnetic magnet is placed inside a tilted microcoil as shown in Figure 2, a magnetic force torque will be generated on the magnet and a torque *T* proportional to magnetic force *F* is exerted on the magnet given by [11].

(1) T=V×|M×H|=VMHsinα∝F

where, the volume of the magnet is *V*, *M* is the magnetization intensity. *α* is the angle between magnetization direction and external magnetic field. Here, it is equal to the value of the tilt-angle. *H* is the magnetic field intensity generated by tilted microcoil. Generally, the smaller the coil pitch, the greater the magnetic field intensity.

**Figure 3:**
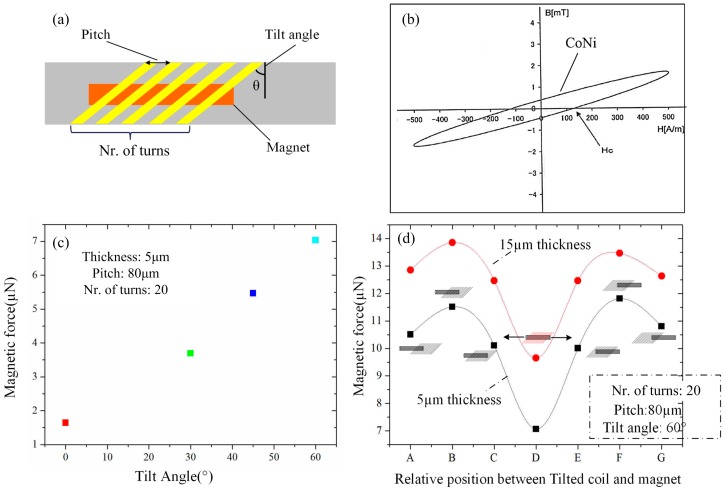
Parameters of designed microcoil and simulated results of magnetic force. (**a**) Key parameters of tilted microcoil and magnet; (**b**) B-H curve of CoNi magnet used; (**c**) Simulated magnetic force with different tilt-angles; (**d**) Simulated magnetic forces with relative positions between coil and magnet. (Nr. means number in the figure).

The key parameters of designed tilted microcoil are shown in Figure 3a, including coil pitch, tilt-angle and turns. ANSYS MAXWELL software (V16.0, Ansys Inc., Canonsburg, PA, USA) was used for simulating the magnetic force, as well as providing us a convenient method and relative precise result. Coil pitch is a decisive factor for magnetic field intensity. Here, the coil pitch of 80 µm is designed in order to provide our fabrication with a greater ability to achieve greater magnetic field intensity. The simulation model consists of two parts: tilted microcoil model and magnet model. The tilted microcoil model was built with different parameters, such as tilt-angle, coil pitch and turns. The material property of the microcoil model is copper, which is applied with 50 mA AC power. On the other hand, a 3 mm length and 600 µm diameter magnet is established, which is made of Cobalt Nickle, whose test coercive field strength is shown in Figure 3b. 

Figure 3c shows that the magnetic force gradually rises as the tilt-angle increases. This is consistent with theoretical torque calculation, where the torque also increases with the angle *α*. However, the tilt-angle is limited in our present fabrication process, and a larger tilt-angle (more than 60°) is difficult to realize due to the insufficient running accuracy of the stage and underexposure. In the present work, a microcoil with a 60° tilt-angle is fabricated in order to obtain, to the best of our ability, a large magnetic force. 

Subsequently, the effect of the location of the magnet on the output magnetic driving force has been taken into consideration in detail. As is indicated in Figure 3d, the magnetic force was simulated at different locations between the tilted microcoil and the magnet (from position A to position E). In the simulation result, the maximum force was obtained at location B or F, where half of the magnet is placed outside the tilted microcoil. It was also simulated in two coils with different thicknesses of 5 µm and 15 µm, respectively. The results show a similar changing trend. 

**Figure 4:**
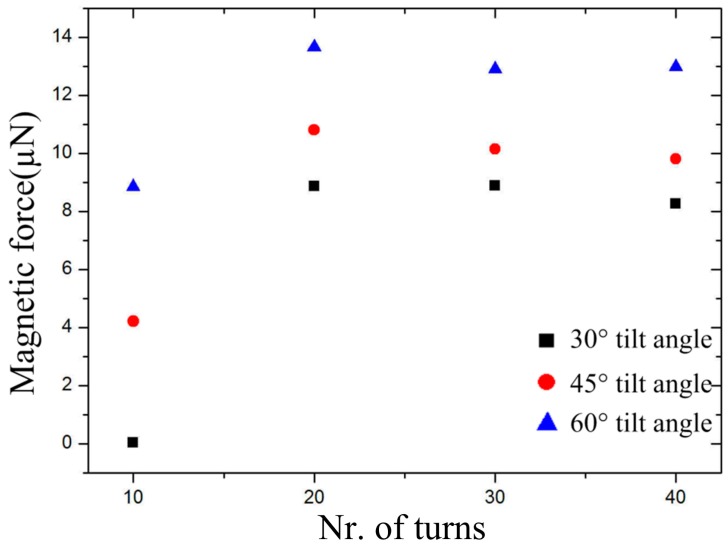
Simulated magnetic force with increasing turns (from 10 to 40) with tilt-angle of 30°, 45° and 60°, respectively.

Next, the magnetic force with an increasing number of turns (ranging from 10 to 40) and tilt-angles of 30°, 40° and 60° was also simulated, as shown in Figure 4. The magnetic force induced by 10 turns is very small, because the magnet is inadequately magnetized. A large magnetic force was obtained in the 60° tilt-angle microcoil with 20 turns. The magnetic force generally remains constant when the turns are equal to or more than 20. This indicates that the magnet will be magnetized to saturation in the tilted magnetic field when half of its volume is placed in a sufficiently long tilted microcoil, such as 20 turns. In addition, an almost identical magnetic force is produced by the tilted coils with 20, 30, and 40 turns, which is independent of turns. These results indicate that the microcoil with 60° tilt-angle exerts a larger magnetic force on the magnet when the other parameters remain constant. The magnetic force continuously changes with the different relative position between the tilted microcoil and the magnet, with the largest one being at the B or F position. The largest magnetic force existed for the microcoil with 20 turns with different tilt-angles when the magnet is placed at the B position. Based on the simulation results and our fabrication capacity, the microcoil with a 60° tilt-angle, 20 turns, and 80 µm coil pitch is ultimately fabricated.

### 2.3. The Modal and Dynamic Analysis

**Figure 5:**
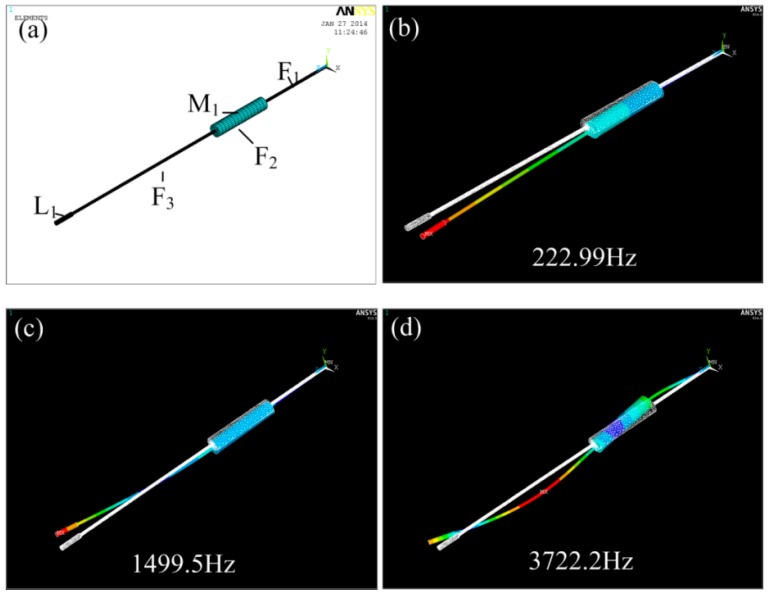
FEA models for modal analysis. (**a**) Whole model for fiber scanner; (**b**–**d**) presented structure deformation and frequency of the first three order resonant modes.

Like all mechanical structures, this fiber scanner has its own resonant frequency, and it achieves the largest amplitude under its resonant frequency. To obtain the resonant frequency of the fiber scanner, modal analysis was conducted by employing commercial ANSYS finite element analysis (FEA) software (V16.0, Ansys Inc., Canonsburg, PA, USA). The geometrical parameters and specifications of the fiber scanner model are given in Table 1. The SOLID 45 element type was chosen, and the SWEEP method was used to mesh the model, as shown in Figure 5a. The first three order modes and corresponding resonant frequencies of the fiber scanner were simulated, as shown in Figure 5b–d, respectively. 

## 3. Fabrication of Tilted Microcoil

### 3.1. Exposure System

**Figure 6:**
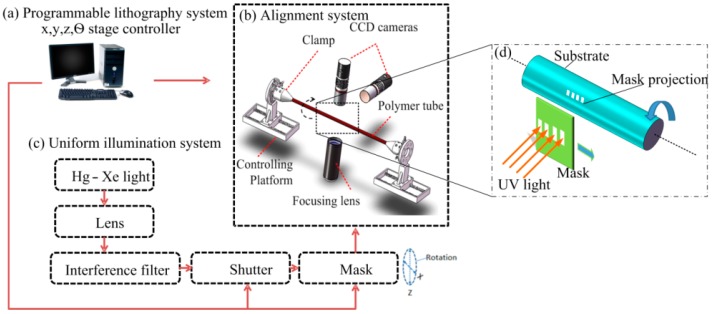
Programmable UV lithography system with alignment for cylindrical substrate: (**a**) programmable lithography system x, y, z, θ-stage controller; (**b**) Charge Coupled Device (CCD) camera alignment system; (**c**) Uniform illumination system; (**d**) Close-up schematic diagram of fabricated sample.

The cylindrical projection lithography system was described in detail in our previous work [12], and its basic setup is shown in Figure 6. The only difference is that the effective exposure area is improved from the previous <20 mm diameter to the present >30 mm diameter circular area, which allows the fabrication of a sufficiently long tilted microcoil with turns of up to 50 in a short time. This exposure equipment mainly consists of three parts: (a) a programmable lithography system with x, y, z, θ-stage controller, (b) a programmed control stage with alignment system, and (c) a uniform illumination system. 

First of all, all the optical elements of the exposure equipment were aligned by a He-Ne laser, after that, the UV light from the illumination source passed through all the aligned optical elements, and the projection of the mask was focused on the surface of the photoresist-coated capillary substrate. Additionally, there are two CCD cameras; one is utilized for observing and adjusting the substrate in order to achieve a stable rotation, the other is used for observation of the alignment state. The substrate and mask were able rotate and move during the whole exposure process. The projection of the mask was able to scan along and around the substrate. Finally, the microcoil pattern was successfully transferred onto the surface of capillary substrate. As a result, the tilted microcoil could be fabricated by rotating the photoresist-coated polyimide capillary, and moving the projection of the mask, as shown in Figure 6d.

### 3.2. Fabrication Process 

**Figure 7:**
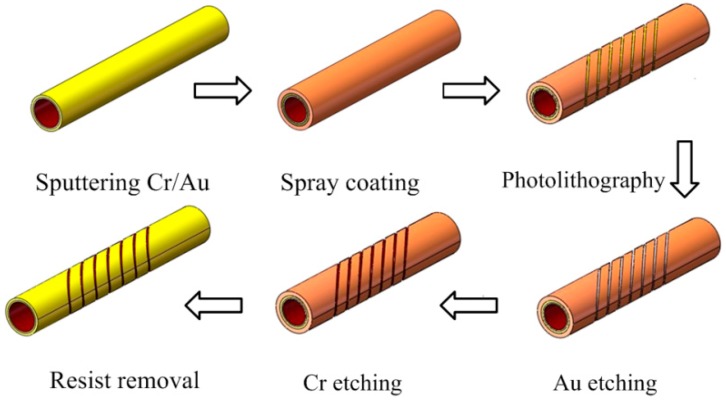
Main fabrication process of the tilted microcoil.

The main fabrication process for the tilted microcoil is shown in Figure 7. At the beginning, a polyimide capillary was sputtered with a 120 nm Cr coating and a 30 nm Au coating. In addition, the polyimide capillary was rotating during the sputtering process to obtain a uniform thin film. This process not only saves time, but also achieves a uniform thickness for the sputtering coating film. This is beneficial to controlling etching time. Next, in order to increase the adhesive force between the Cr and Au layers, a thermal treatment process was employed at 200 °C for 20 min after sputtering. After that, photoresist was coated onto the surface of the samples using the spray-coating system that was developed for preparing thin uniform resist coating on cylindrical substrates [13]. The surface treatment process using a UV ozone treatment unit was carried out before the spray coating. 

The two ends of the sample can be fixed by two clips mounted on the two motors, which can be driven synchronously at a certain rotation speed using the control panel during the spray-coating process. A heater nozzle is mounted next to the spray nozzle to realize real-time heating treatment, which is necessarily employed to obtain a smooth surface without pinholes. Without real-time heating treatment, there are some pinholes on the prepared coating, wavelike surface morphology is also visible, as was verified in our previous research [13]. Some major parameters of the spray-coating process can be independently controlled and programmed, such as the distance between the spray nozzle and the substrate, rotation speed of the sample, scan speed of the spray nozzle, spray cycles, and so on. Here, the distance between the spray nozzle and the substrate is 56 mm. A better surface with few pinholes and particles can be obtained at this distance. Shipley S1830 positive photoresist (Shipley Co. LLC, Marlborough, MA, USA) was employed as the spray-coating photoresist. The solvent was AZ5200 thinner (AZ electronic materials, Luxembourg), which consists of propylene glycol monomethyl ether acetate (PGMEA), i.e., a common solvent for the direct spraying-coating process. Finally, a 4 µm thickness of the photoresist coating was sprayed onto the circumferential surface of the sample. 

For the lithography process, firstly, the sample was loaded on the cylindrical projection exposure equipment, and then it was fixed tightly by two clamps mounted on the two synchronously driven rotation stages. Next, the sample was adjusted so as to remain level and the control program, compiled in advance, was loaded into a personal computer. Finally, the sample was exposed by UV light with the shutter open. A tilted microcoil pattern of the photoresist was realized by controlling the rotation speed of the substrate and the linear movement speed of the mask. After exposure and development of the photoresist, the Cr and Au coatings were etched by wet etching. On the basis of the thickness of the coating and the etch rates, the sample was immersed in the Au etching liquid for 25 s, and then put into the Cr etching liquid for 1 min. Next, the photoresist was removed in acetone. Finally, the copper was electroplated on the Au seed-layer of the coil pattern by mask-less electroplating process. The electroplating resolution developed by our lab and Meltex Inc., Japan was employed, in which a kind of novel additive was used to obtain a high quality of electroplated film [14].

## 4. Characterization

### 4.1. Characteristics of Tilted Microcoil Patterns

**Figure 8:**
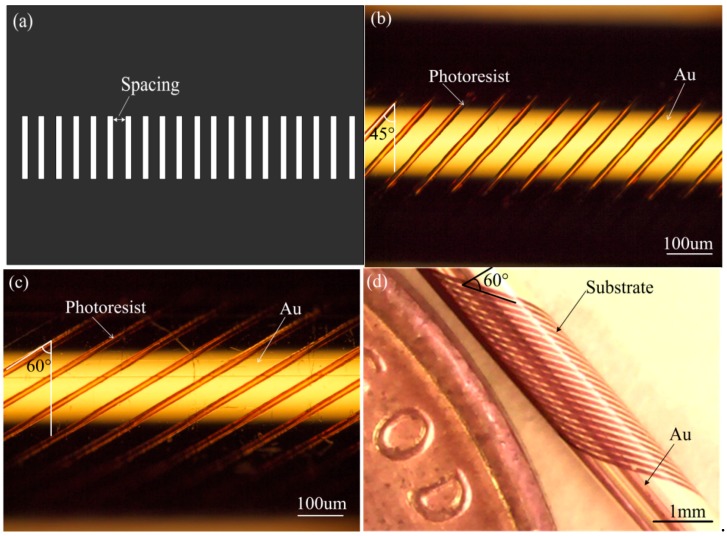
Mask pattern and tilt microcoil pattern. (**a**) Mask pattern with multi-lines; (**b**) Optical image of fabricated microcoil with 45° tilt-angle; (**c**) Optical image of the fabricated microcoil with 60° tilt-angle; (**d**) Photography of 20 turn microcoil with 60° tilt-angle fabricated on the 1 mm diameter ployimide capillary substrate.

The main parameter dimensions of the designed tilted microcoil pattern are decided by the lithography process. Two kinds of masks were used for patterning the tilted microcoil in the exposure process. These are one-line pattern mask and multi-line pattern mask. To save exposure time, multi-line pattern mask is the best selection, where there are 20 lines in the mask, each of which is 40 × 170 μm^2^, and the spacing between lines is 180 µm and 310 µm for 45° and 60° tilt-angles, respectively, as shown in Figure 8a. 

According to our design and calculations, the spacings 180 µm and 310 µm are the critical distances for using 40 × 170 μm^2^ lines to fabricate 45° tilt-angle and 60° tilt-angle microcoils, respectively, while avoiding overlap exposure. The tilted microcoil pattern can be realized by controlling the rotation speed of the substrate and the linear movement speed of the mask synchronously, as mentioned above. The distances of linear movement can be decided according to the tilt-angle, are are 3180 µm and 5444 µm for 45° tilt-angle and 60° tilt-angle microcoils, respectively. The exposure time is selected based on the size of the line pattern. In our experiment, 10 min, 20 min, 30 min, 40 min and 50 min were attempted successively. For the mask with a 40 × 170 µm^2^ line pattern, 40 min exposure time was sufficient for our designed microcoils. The rotation speed of the substrate and the movement speed of the mask are also closely related to the exposure time, and can be calculated and evaluated based on the exposure time. Mask movement is separated into two parts: forward movement, for 0°–180° rotation of the substrate; and backward movement, for 180°–360° rotation. The distance of forward movement is 3180 µm for 45° tilt-angle microcoils and 5444 µm for 60° tilt-angle microcoils. However, to obtain a continuous coil line, the distance for backward movement is 3000 µm for 45° tilt-angle microcoils and 5134 µm for 60° tilt-angle microcoils, when considering the subtraction between movement distance and line spacing on the mask. The rotation speed and movement speed are decided as follows: forward movement speed, backward movement speed and rotation speed for the 45° tilt-angle microcoil are approximately 2.65 µm/s, 2.50 µm/s and 0.15 deg/s, respectively. Forward movement speed, backward movement speed and rotation speed for the 60° tilt-angle microcoil are approximately 4.54 µm/s, 4.28 µm/s and 0.15 deg/s, respectively.

A representative optical image of the photoresist pattern of the microcoil with 45° tilt-angle fabricated on the polyimide capillary by the cylindrical projection lithography technique is shown in Figure 8b. The photoresist pattern of microcoil with a tilt-angle of 45° was patterned with 10 ± 0.2 µm coil width, 78.5 ± 0.5 µm coil pitch and 20 turns. In addition, the photoresist pattern microcoil with tilt-angle 60° was also successfully patterned with 10 ± 0.2 µm coil width, 81.5 ± 0.5 µm coil pitch and 20 turns, as shown in Figure 8c. A photograph of the 20 turn microcoil with 60° tilt-angle on the 1 mm diameter polyimide capillary after Au etching, Cr etching and photoresist removal is presented in Figure 8d.

### 4.2. Characteristics of Electroplated Tilted Microcoil

**Figure 9:**
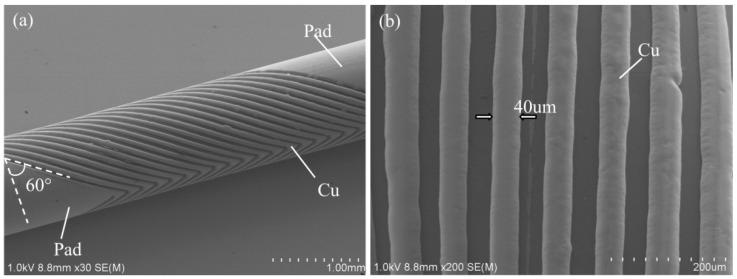

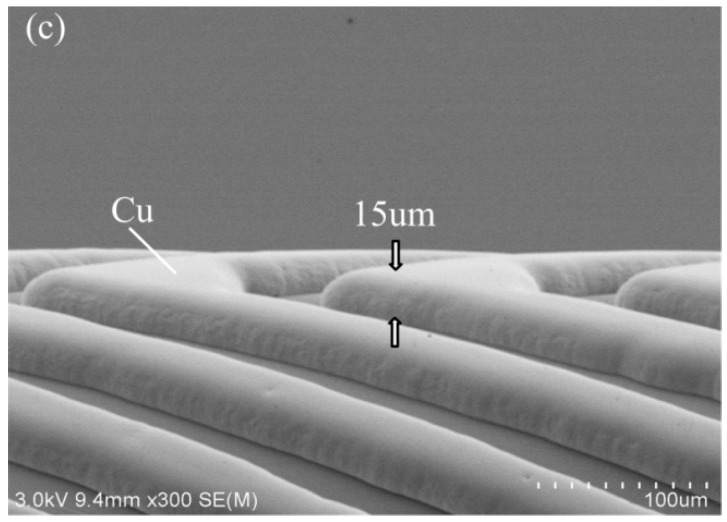
SEMs of the electroplated tilted microcoil. (**a**) Intact coil; (**b**) line width and spacing; (**c**) thickness of Cu microcoil.

The SEMs of the electroplated copper microcoil are shown in Figure 9a. Copper electroplating would be produced directly on the Au coil line used as the seed layer without photoresist mold. In addition, the Cu coil line grows along the Au coil line vertically, and slightly expands both sides during the electroplating. In our case, the Cu coil line width finally increases to 40 µm, and the thickness grows to 15 µm based on a 10 µm Au coil line. The microcoil with a tilt-angle of 60°, line width of 40 µm, and thickness of 15 µm was successfully obtained, as shown in Figure 9b,c.

## 5. Conclusions

We proposed an ultra-thin single-fiber scanner that is electromagnetically driven by tilted microcoils. It can be fabricated by a specific MEMS process. The parameters of the tilted microcoil were designated as 60° tilt-angle, 80 µm coil pitch, and 20 turns based on the simulation and optimization results for magnetic force. The modal response of the designed scanner was simulated. The tilted microcoils were fabricated on a polyimide capillary surface by the developed MEMS-based fabrication process. The process includes spray coating, cylindrical projection lithography, and mask-less electroplating micromachining. In particular, the photoresist pattern of the microcoil has been obtained successfully and with high resolution using this lithography method. The dimensions of the two fabricated tilted microcoils were as follows: 45° tilt-angle, 10 µm ± 0.2 µm line width, 78.5 ± 0.5µm coil pitch and 20 turns; and 65° tilt-angle, 10 µm ± 0.2 line width, 81.5 ± 0.5 µm coil pitch and 20 turns. Finally, a direct mask-less electroplating process was used to obtain a 15 µm thick copper microcoil.

## Figures and Tables

**Table 1:** Geometric parameters and material properties for the fiber scanner model.

Components	Geometric Parameters and Material Property	Values
Optical fiber	Elastic Modulus E_f_	1.72 GPa
	Poisson ratio R_f_	0.17
	Density ρ_f_	2200 Kg/m^2^
	Length F_1_, F_2_, F_3_	4 mm, 3 mm, 6 mm
	Diameter D_f_	125 µm
Magnet	Elastic Modulus E_m_	210 GPa
	Poisson ratio R_m_	0.31
	Density ρ_m_	8500 Kg/m^2^
	Length M_1_	3 mm
	Diameter D_m_	600 µm
Lens	Elastic Modulus E_l_	1.72 GPa
	Poisson ratio R_l_	0.17
	Density ρ_l_	2200 Kg/m^2^
	Length L_1_	2 mm
	Diameter D_l_	1 mm
